# Prolonged Effects of Acute Stress on Decision-Making under Risk: A Human Psychophysiological Study

**DOI:** 10.3389/fnhum.2016.00444

**Published:** 2016-09-13

**Authors:** Kaori Yamakawa, Hideki Ohira, Masahiro Matsunaga, Tokiko Isowa

**Affiliations:** ^1^Department of Psychology, Graduate School of Environmental Studies, Nagoya UniversityNagoya, Japan; ^2^Department of Psychology, School of Humanities, Tokaigakuen UniversityNagoya, Japan; ^3^Department of Health and Psychosocial Medicine, Aichi Medical University School of MedicineNagakute, Japan; ^4^Department of Nursing, Graduate School of Medicine, Mie UniversityTsu, Japan

**Keywords:** acute stress, decision-making, risk, reflection effect, cortisol

## Abstract

This study investigates the prolonged effects of physiological responses induced by acute stress on risk-taking in decision-making. Participants were divided into a Stress group (*N* = 14) and a Control group (*N* = 12). The Trier Social Stress Test was administered as an acute stressor, and reading was administered as a control task; thereafter, participants performed a decision-making task in which they needed to choose a sure option or a gamble option in Gain and Loss frame trials 2 h after (non-) exposure to the stressor. Increased cortisol, adrenaline, heart rate (HR), and subjective stress levels validated acute stress manipulation. Stressed participants made fewer risky choices only in the Gain domain, whereas no effect of stress was shown in the Loss domain. Deceleration of HR reflecting attention was greater for Gains compared with Losses only in the Stress group. Risk avoidance was determined by increased levels of cortisol caused by acute stress. These results suggest that processes regarding glucocorticoid might be involved in the prolonged effects of acute stress on the evaluation of risks and the monitoring of outcomes in decision-making.

## Introduction

Many important decisions, particularly in economics, are made under stress. For example, stock dealers must make financial decisions under time and psychological pressures while facing difficulties in the workplace and in interpersonal relationships. Influences of acute stress on decision-making have been supported by accumulated empirical findings (Starcke and Brand, 2012) and by observations of real financial working conditions, for instance, in stock trading centers (Coates and Herbert, 2008). However, the investigation of physiological mechanisms underlying influences of acute stress on decision-making is only in its primary stages.

Previous human psychological studies have generally reported facilitation of risk-taking in several kinds of decision-making tasks after exposure to acute stress (e.g., Lighthall et al., 2009; Galvan and McGlennen, 2012; Morgado et al., 2015). However, the findings from previous studies are complicated and mixed. For example, acute stress facilitated risk avoidance when decisions involved gains but facilitated risk preference when decisions involved losses, resulting in a strengthened “reflection effect,” i.e., greater preference for risky options linked with losses rather than gains (Kahneman and Tversky, 1979; Porcelli and Delgado, 2009). However, the opposite result, that acute stress led to risk avoidance of losses, has also been reported (Clark et al., 2012). Furthermore, some studies found null effects of acute stress on risk-taking in decision-making (Lempert et al., 2012; Gathmann et al., 2014).

A possible cause of inconsistency of previous findings is temporal characteristics of stress responses. Immediately after exposure to acute stress, responses in several biological systems progress in parallel, for example, the sympatho-adrenal medullary (SAM) system (Kvetnansky et al., 2009), the hypothalamus–pituitary–adrenal (HPA) axis (Kudielka and Kirshbaum, 2004), and inflammation (Dantzer, 2006). Temporal changes in levels of activity in these systems should inevitably show complications because such systems are mutually linked by positive- and negative-feedback pathways (Dantzer, 2006). In addition, because substances involved in these systems, such as catecholamine, corticosteroids, and cytokines, have facilitating and inhibiting influences on the central nervous system related to cognitive and emotional processes underlying decision-making (Preston et al., 2007; Wolf, 2009; Starcke and Brand, 2012), the effects of acute stress on risk-taking should fluctuate and be unstable in the period immediately after the onset of acute stress. Indeed, risk avoidance was first observed when the decision-making task was performed 5 or 18 min after acute stress, but risk-taking was promoted when a decision-making task was performed 28 min after stress (Pabst et al., 2013).

Previous literature has shown that, even hours after the induction of stress, acute stress can robustly affect many facets of higher-order cognitive functions, including emotional memory (Diamond et al., 2007), selective attention to emotional stimuli (Henckens et al., 2012), working memory (Henckens et al., 2011), and altruistic punishment in an economic game (Vinkers et al., 2013). These findings led us to infer the possibility of acute stress also affecting decision-making under risk in longer time ranges. If this inference is correct, an important question regarding underlying biological mechanisms is whether the strength of physiological responses (HPA and SAM responses) (Diamond et al., 2007) to acute stress at an early stage can determine the strength of later stress effects on bias toward risk-taking in decision-making. The present study explored this issue with an experiment measuring typical physiological indices of stress and risky choices in a lottery decision-making task performed 2 h after exposure to acute stress.

Furthermore, we examined two issues of psychological mechanisms through which acute stress can affect later decision-making. The first issue is whether effects of acute stress on risk-taking occur through experiential or descriptive processes (Buckert et al., 2014). Namely, acute stress might affect psychological responses to hit-and-miss feedback; therefore, stress can bias future risk-taking through learning processes (the experience account). On the other hand, acute stress might affect evaluations of the magnitude of reward and probability and can thus bias risk-taking (the description account). To examine this issue, we tested dependency of risky choice rates on previous outcomes. If the experiential account is correct, a probability of a risky choice would increase just after receiving a reward and decrease just after losing a reward (Win-Stay, Lose-Shift). If the description account is correct, a probability of a risky choice would be rather independent of previous outcomes. In addition, we measured the transient deceleration of heart rate (HR) as an orienting response, which is a typical physiological response to feedback signals in decision-making (Bradley, 2009; Osumi and Ohira, 2009). If the experiential account is correct, HR deceleration should correlate with risk-taking after exposure to acute stress. If the description account is correct, acute stress should independently affect HR deceleration and risk-taking. The second issue is whether alterations in risk-taking caused by acute stress are based on habit action or goal-directed action (Schwabe et al., 2008; Dias-Ferreira et al., 2009). Acute stress can make habit action more dominant; therefore, individuals might habitually prefer or avoid risk without deliberating over expected gains or losses. Contrarily, acute stress can facilitate individuals’ strategic deliberation in preferring or avoiding risk to satisfy their inner goals. To examine this issue, we manipulated expected values (EV) of a safer and a riskier option in each decision-making trial. In some trials, the riskier option provided higher EV than the safer option, while in other trials, the EV of the risker option was lower than that of the safer option, or EVs of both options were identical. If acute stress makes habit action dominant, participants should show consistent within-individual tendencies of risk preference or risk avoidance regardless of differences of EVs. However, if acute stress facilitates goal-directed action, participants should become more sensitive to EVs of options, and they should make risk-taking or risk-avoiding choices depending on relative comparisons of the EVs of options.

## Materials and Methods

### Participants

In the present study, 28 Japanese male undergraduates at Nagoya University participated (age range 18–22 years; mean = 19.92; *SD* = 1.20). They were randomly assigned to a Stress group or a Control group. This sample size was determined according to *a priori* analysis of statistical power using G^∗^power 3, version 3. 1. 9. 2 (Faul et al., 2007). A sample size adequate to detect the effect size reported by Pabst et al. (2013), indicating significant effects of acute stress on risky choice, was estimated as *N* = 24 (alpha error = 0.05; 1-beta error = 0.95). Two participants in the Stress group were excluded from analysis because of technical problems in data collection. No participants suffered from any chronic illnesses, and none took any medications. Participants were advised not to smoke or drink alcohol on days they participated in the experiments. The Ethics Committee of Nagoya University approved the study (No.: 315, date: 25th June, 2012), and its methods were conducted according to approved guidance for human subjects. All participants signed an informed consent before participating in the study.

### Decision-Making Task

**FIGURE 1:**
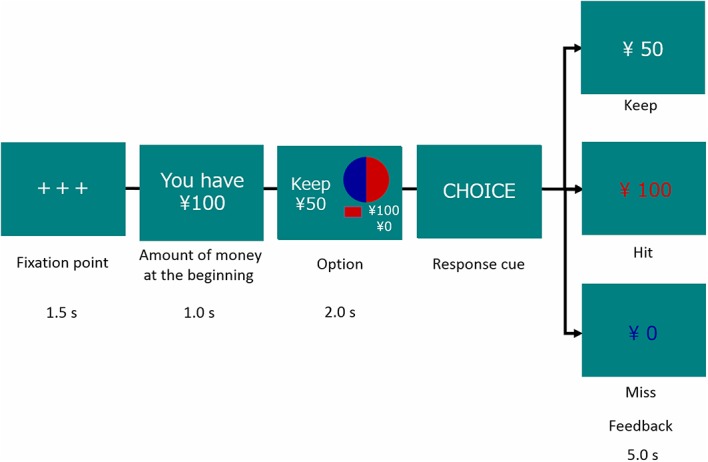
**Decision-making task**.

At the beginning of each trial, participants were shown a message indicating a starting amount of money. They had to choose a sure or a gamble option for each trial. The sure option meant keeping the amount of money given at the beginning in Gain frame trials and losing the amount given at the beginning in Loss frame trials. The gamble option was shown as a pie chart depicting the probability of Hit (red) or Miss (blue) and the amount of monetary reward or loss (see **Figure 1**). Options were presented for 2 s, followed by a response cue to prompt participants to choose. After the choice, a feedback signal (Keep, Hit, or Miss) was indicated.

In this task, we manipulated EV to assess decision-makers’ sensitivity and adaptability to contingencies making a choice advantageous or disadvantageous. For this manipulation, we set conditions at three different EV levels between gamble and sure options: a large EV gamble with a larger EV of the gamble option than of the sure option; a small EV gamble with a smaller EV of the gamble option than of the sure option; and an equal EV gamble with the same EV of the gamble option as of the sure option. Therefore, independent variables in this task were Domain (Gain or Loss) and EV of gamble option (Large, Equal, and Small). Additionally, we manipulated the probability of the hit outcomes when the gamble option was chosen (see Supplement 1). Gain and Loss trials were presented as separate blocks, with counterbalanced orders across participants in each group. Within a block, conditions of the EV gamble option were presented randomly.

### Procedures

Experimental sessions started at either 09:00 a.m. or 12:00 p.m. and lasted for 4 h. To control effects of diurnal variations in cortisol secretion, numbers of participants allocated into early and late experimental sessions were counterbalanced between Stress and Control groups. Participants were instructed to eat a light breakfast on the morning of the experiment, but not to drink caffeinated beverages. Participants suffering from an infectious illness within 2 weeks of the experiment were rescheduled.

**FIGURE 2:**
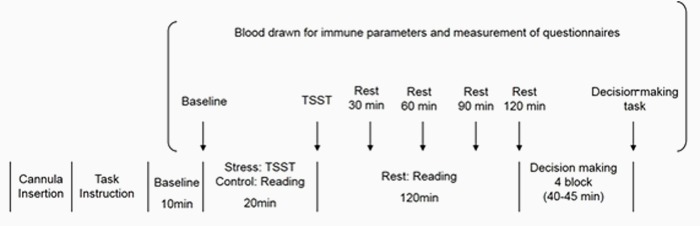
**Experimental protocol of the present study**.

The timeline of the experimental session for both groups is presented in **Figure 2**. After participants entered the experimental chamber, a cannula was inserted into their non-dominant forearm vein. Next, electrodes for electrocardiographic measurements were attached to the same arm. After the first rest period of 10 min, the first blood sample was taken as a baseline, and participants were asked to rate subjectively their intensity of stress. Thereafter, instructions for the “Trier Social Stress Test” (TSST) (Kirschbaum et al., 1993) were provided. Following this, participants were given 10 min to prepare for their speech. They were then exposed to a simulated interview (5 min) in front of a video camera and conducted by two interviewers, followed by a mental arithmetic task (5 min). Immediately after the task, a second blood sample was taken, and participants again subjectively rated their stress. Participants read newspapers during the 120-min rest period. After each rest period (30, 60, 90, or 120 min after completing the TSST), the third, fourth, and fifth blood samples were taken; again, participants rated their stress subjectively. Finally, instructions for the decision-making task were given, and participants completed the decision-making task after several practice trials. Cardiodynamic activity was measured continuously throughout the experimental session. After the procedure ended, electrodes and the cannula were removed, and participants were fully debriefed and thanked. The experimental session in the Control group was identical to that in the Stress group, except that participants read newspapers for 20 min instead of focusing on the TSST.

### Psychological and Behavioral Measures

Participants were asked to evaluate intensity of their stress on a visual analog scale (0–100%) after the baseline period, the TSST task period, each rest period, and the decision-making period. For a behavioral index of decision-making, we calculated rates of gamble choices in each condition of the decision-making task. As a trial-wise behavioral index to evaluate dependency of risk-taking on a previous outcome, we calculated the rates of gamble choices after each outcome (e.g., keep, gamble-hit, and gamble-miss) in each condition. In addition, conditional entropy was calculated as a global index of dependency of risk-taking on previous outcomes (Ohira et al., 2013a, 2014). First, we determined a conditional probability of an action (*a*) under a state (*S*). Here, the action is a choice of gamble or keep. The state is an outcome (keep, gamble-hit, or gamble-miss) in the previous trial. Thus, the conditional probability *P*(*a*|*S*) is calculated as follows:

$$P(a|\;S)=\frac{Num(a|\;S)+c}{\Sigma_{k}\{Num(k|\;S+c)\}},$$

where *Num(a|S)* is the number of choices of gamble or keep (*a*) under a state *S*, and *Num(k|S)* is the number of total choices *k* under a state *S*. The constant *c* was introduced to stabilize the calculated probability and was fixed to 1 here. Then, entropy H was estimated as follows:

$$H=-\frac{1}{N}\underset{S}{\Sigma }\underset{a}{\Sigma }P(a|S)\log_{2}P(a|S),$$

where *N* is a number of states *S*. The value of entropy *H* was standardized from 0 to 1 by dividing by *N* (here, *N* = 3). Conditional entropy calculated by this formula reflects the degree of deviation from dependence of a choice on the outcome of the previous trial. For example, if a participant more often chooses gamble when hit was given in the previous trial and chooses keep when miss was given in the previous trial (Win-Stay, Lose-Shift), then *H* will be smaller (approaching 0). This pattern of decision-making can be regarded as more dependent on previous outcomes and thus more experiential. Conversely, as a participant more often chooses gamble or keep totally independently from the previous trial’s outcome, then entropy *H* will be larger (approaching 1). This pattern of decision-making is regarded as more independent from previous outcomes and probably stochastically determined based on an inner standard and is thus more descriptive.

### Cortisol and Adrenaline Measurement

Blood samples were collected in EDTA tubes and centrifuged at 3,000 rpm for 10 min to measure cortisol levels in plasma. Plasma was then separated and stored at -80°C until analysis. The plasma cortisol concentration was measured using a cortisol ELISA kit (Oxford Biochemical Research Inc., Oxford, MI, USA). The intra-assay coefficient of variation was 3.4–3.7%, and the inter-assay coefficient of variation was 3.8–6.4%. The limit of detection was 0.3 μg/ml. To determine adrenaline levels, blood samples were collected in serum separator tubes and centrifuged for 15 min. Serum was removed and then kept at -80°C until analysis. The concentration of adrenaline in serum was measured using an HPLC-electrochemical detector (ECD) (CoulArray; ESA Biosciences, Inc.). The inter-assay coefficient of variation was less than 7.0%. The limit of detection was 0.1 ng/ml.

### Cardiac Measure

Cardiodynamic activity was recorded with an electrocardiogram (ECG) at 500 Hz, using the MP 100 system (Biopac Systems Inc., Goleta, CA, USA) and Ag/AgCL electrodes on the extremities. Analysis of ECG waveforms was performed using AcqKnowledge software for MP 100 (Biopac System, Santa Barbara, CA, USA). After rejection of artifacts in ECG waveforms, HR and inter-beat-interval data were derived during the baseline period (10 min), the stress tasks period (15–20 min), each rest period (30 min), and the decision-making period (40–45 min). To analyze cardiac data in the decision-making task, inter-beat intervals were obtained from deviations between R-waves and converted into beats per minute (bpm). HR in bpm was averaged in half-second intervals and deviated from the 1-s baseline preceding feedback onset. Initial deceleration was assessed as a minimum value in 0–3 s of the feedback presentation period in each trial. As is known, HR deceleration reflects attentional orienting governed by parasympathetic activity (Bradley, 2009; Osumi and Ohira, 2009). For this reason, we focused on examining outcome-related HR reactivity induced by feedback signals of hit and miss.

### Experimental Design and Statistical Analyses

To certify that the TSST evoked acute stress responses, data showing HR, cortisol, adrenaline, and subjective stress levels during the TSST were analyzed using 2 (Group: Stress vs. Control) × 7 (Period: Baseline, TSST, Rest_30__min_, Rest_60__min_, Rest_90__min,_ Rest_120__min_ periods, Decision-making task) repeated measures analyses of variance (ANOVAs). Group was a between-participant factor, and Period was a within-participant factor. To analyze decision-making performance, rates of gamble choice, and conditional entropy were administrated for a 2 (Group: Stress vs. Control) × 2 (Domain: Gain vs. Loss) × 3 (EV level of gamble option: Large_,_ Equal, and Small) repeated measures ANOVA. Group was a between-participant factor, and Domain and EV level were within-participant factors. Furthermore, for rating gamble choice per previous outcome, a 2 (Group: Stress vs. Control) × 2 (Domain: Gain vs. Loss) × 3 (EV level of gamble option: Large, Equal, and Small) × 3 (Previous outcome: Keep, Gamble-hit, Gamble-miss) repeated measures ANOVA was conducted. Previous outcome was a within-participant factor. Cardiac data for feedback in the decision-making task were analyzed using a 2 (Group: Stress vs. Control) × 2 (Domain: Gain vs. Loss) × 2 (Outcome: Hit vs. Miss) × 3 (EV level of gamble option: Large_,_ Equal, and Small) repeated measures ANOVA. Group was a between-participant factor and Domain, Outcome, and EV level were within-participant factors. The Greenhouse–Geisser epsilon correction factor 𝜀 (Jennings and Wood, 1976), was used where appropriate. When significant interactions were found in ANOVAs, *post hoc* analyses using Bonferroni tests (*p* < 0.05) were conducted to examine which combinations of data points differed significantly.

To examine the association between physiological acute stress responses and decision-making, structural equation modeling (SEM) was conducted using physiological parameters as predictors for rates of gamble choice in decision-making (for details, see Results, Association between physiological parameters and decision-making). Considering the sample size of the present study, overall model fit was assessed using chi-square/degree of freedom (df) ratio, goodness-of-fit index (GFI), and root mean square error of approximation (RMSEA). A chi-square/df ratio ≤0.20, a GFI ≥ 0.95, and an RMSEA ≤ 0.05 are considered the standard of a good fit (West et al., 2012).

## Results

### Manipulation Check of Acute Stress

**FIGURE 3:**
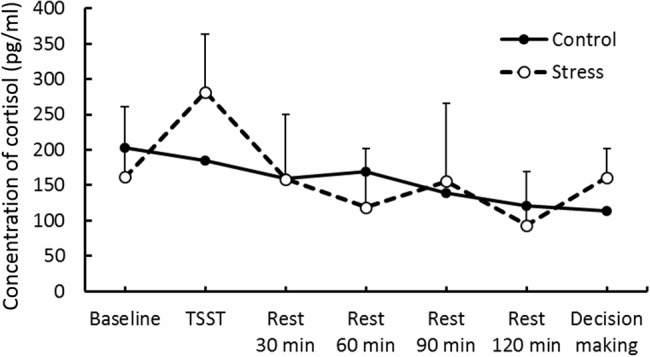
**Cortisol concentration in each group.** Error bars indicate standard errors of means.

**FIGURE 4:**
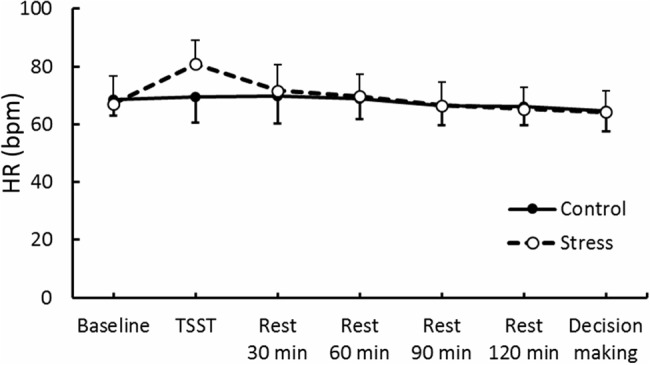
**Heart rate (HR) through experimental sessions in each group.** Error bars indicate standard errors of mean.

**FIGURE 5:**
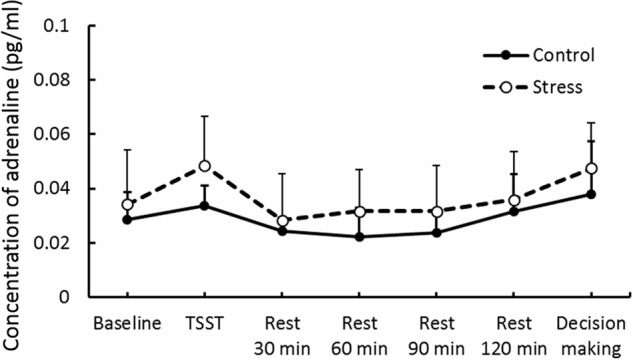
**Adrenaline concentration in each group.** Error bars indicate standard errors of means.

Results of psychological data are presented in **Table 1**. A repeated measures ANOVA revealed significant interaction between Group and Period, [*F*(3.53,84.74) = 7.70, *p* < 0.05, $\eta_{p}^{2}$ = 0.25]. *Post hoc* analyses (*p* < 0.05) indicated that perception of stress after the TSST task was higher than that at baseline in the Stress group. Significant interaction between Group and Period was observed for cortisol, [*F*(3.88,93.15) = 4.46, *p* < 0.05, $\eta_{p}^{2}$ = 0.16] (**Figure 3**). The cortisol level significantly increased after the TSST task compared with the level at baseline or levels during rest periods in the Stress group, but not in the Control group (*p* < 0.05). Significant interaction between Group and Period for HR was found [*F*(3.31,79.45) = 12.93, *p* < 0.05, $\eta_{p}^{2}$ = 0.35] (**Figure 4**). Further analyses (*p* < 0.05) indicated that HR increase in the Stress group was greater during speech tasks compared with the Control group. As an index of activation of the sympathetic nervous system, adrenaline concentration showed significant interaction between Group and Period [*F*(3.83,91.93) = 6.50, *p* < 0.05, $\eta_{p}^{2}$ = 0.21] (**Figure 5**). *Post hoc* analyses (*p* < 0.05) indicated that after the TSST task, adrenaline level in the Stress group was higher than that of the Control group, but no difference was observed during rest periods. All these indices consistently clarified that the stress task in this study elicited typical, robust psychological and physiological (SAM and HPA) acute stress responses.

**Table 1:** Means (Standard Error of Mean) in the intensity of stress and results of ANCOVA.

	Baseline	TSST	Rest 30 min	Rest 60 min	Rest 90 min	Rest 120 min	Decision- making task
Stress	2.05	5.47^∗^	2.24	1.78	2.28	2.37	3.97
	(1.95)	(2.37)	(1.24)	(1.29)	(1.77)	(2.12)	(1.41)
Control	1.03	1.19	1.63	1.57	1.36	1.42	3.21
	(1.12)	(0.92)	(0.80)	(0.73)	(0.67)	(0.77)	(1.31)

*^∗^Significant differences from baseline value in each group (p < 0.05).*

### Effects of Stress on Decision-Making

#### Gamble Choice and Conditional Entropy

**FIGURE 6:**
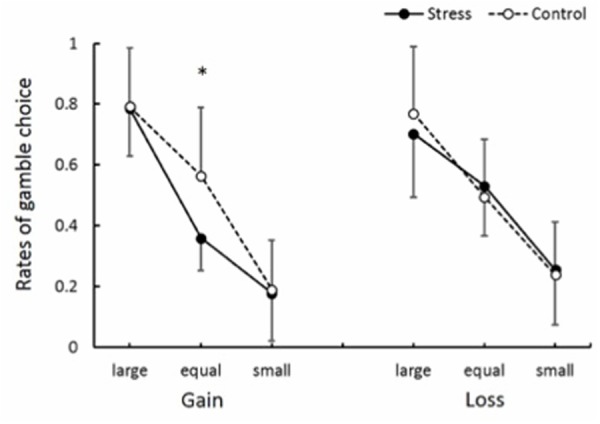
**Rates of gamble choice.** ^∗^Significant difference between groups (*p* < 0.05).

Gamble choice rates in experimental conditions are shown in **Figure 6**. There was significant interaction between Group, Domain, and EV level [*F*(2,48) = 4.66, *p* < 0.05, $\eta_{p}^{2}$ = 0.16]. *Post hoc* comparisons (*p* < 0.05) revealed that the gamble choice rate in the Equal EV level for the Gain domain in the Stress group was smaller than that in the Control group. The gamble choice rates for each previous outcome are summarized in **Table 2**. Naturally, a main effect of EV level was significant [*F*(1.54,37) = 86.59, *p* < 0.001, $\eta_{p}^{2}$ = 0.78], suggesting the gamble option was chosen more as the EV of the gamble option was higher than that of the safe option. Also, a main effect of Previous outcome was significant [*F*(1.48,35.59) = 20.94, *p* < 0.001, $\eta_{p}^{2}$ = 0.47], and *post hoc* comparisons (*p* < 0.05) indicated that the gamble option choice was less after a participant chose the sure option compared to after he chose the gamble option. Furthermore, an interaction between Previous outcome and EV level was significant [*F*(2.55,61.19) = 2.92, *p* < 0.05, $\eta_{p}^{2}$ = 0.11]. *Post hoc* comparisons (*p* < 0.05) revealed that the gamble choice rate after a keep trial was smaller than that after a hit or a miss trial when the EV of the gamble choice was smaller than or equal to the sure option. Significant effects regarding Group (Stress vs. Control) were not observed. As shown in **Table 3**, overall, conditional entropy showed relatively higher values (*H* > 0.50) in all experimental conditions. Neither main effects nor interactions indicated significant levels (*F* < 1.60); thus, acute stress showed no effect on conditional entropy.

**Table 2:** Means (Standard Error of Mean) in the rates of gamble choice per each previous outcome.

	After lose						After win						After keep					
	Gain			Loss			Gain			Loss			Gain			Loss		
	Small	Euqal	Large	Small	Euqal	Large	Small	Euqal	Large	Small	Euqal	Large	Small	Euqal	Large	Small	Euqal	Large
Stress	0.38 (0.10)	0.46 (0.11)	0.79 (0.15)	0.36 (0.16)	0.46 (0.14)	0.68 (0.20)	0.42 (0.16)	0.51 (0.19)	0.67 (0.21)	0.42 (0.15)	0.54 (0.12)	0.70 (0.20)	0.19 (0.16)	0.27 (0.22)	0.66 (0.10)	0.27 (0.21)	0.38 (0.22)	0.60 (0.19)
Control	0.46 (0.14)	0.44 (0.18)	0.78 (0.18)	0.39 (0.10)	0.52 (0.19)	0.78 (0.16)	0.33 (0.14)	0.54 (0.19)	0.72 (0.22)	0.41 (0.15)	0.50 (0.15)	0.71 (0.21)	0.20 (0.17)	0.34 (0.23)	0.63 (0.14)	0.26 (0.21)	0.36 (0.24)	0.60 (0.19)

**Table 3:** Means (Standard Error of Mean) in the conditional entropy showed relatively higher values (*H* > 0.50) in all experimental conditions.

	Gain			Loss		
	Small	Euqal	Large	Small	Euqal	Large
Stress	0.57 (0.27)	0.51 (0.30)	0.65 (0.17)	0.62 (0.25)	0.67 (0.25)	0.70 (0.22)
Control	0.55 (0.25)	0.53 (0.26)	0.60 (0.23)	0.64 (0.28)	0.59 (0.26)	0.57 (0.24)

#### Cardiac Responses to Gamble Outcomes in Decision-Making

**FIGURE 7:**
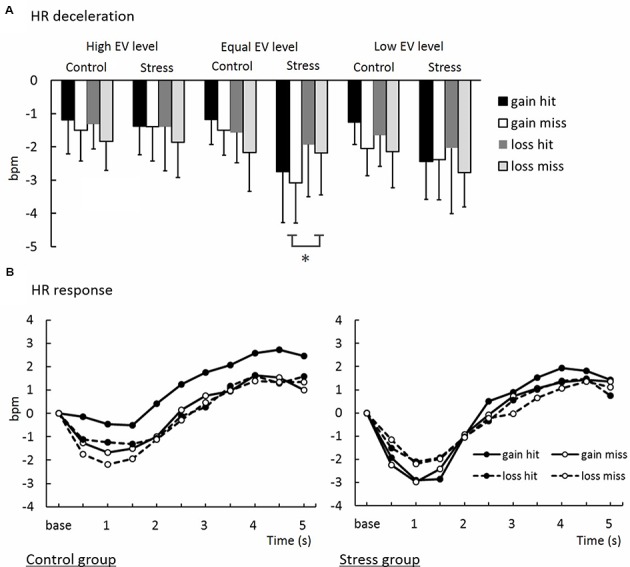
**(A)** Heart rate deceleration in decision-making task. ^∗^Significant difference between groups (*p* < 0.05). **(B)** Waveforms of time-series variations of HR deceleration.

The means of magnitudes of HR deceleration from baseline (HR values for 1 s preceeding feedback onset; see Methods, Cardiac measure) are summarized in **Figure 7A**. An ANOVA for magnitudes of HR deceleration revealed a significant interaction between Group, Domain, and EV [*F*(7.78,41.42) = 4.13, *p* < 0.05, $\eta_{p}^{2}$ = 0.02]. *Post hoc* comparisons (*p* < 0.05) revealed that HR in the Equal EV level for the Gain domain in the Stress group decelerated more than that in the Control group. As shown in **Figure 7B**, HR time-locked to gamble outcomes in the Equal EV level showed deceleration that can be interpreted as a typical orienting response. In contrast to gamble option choice, HR deceleration to outcome of a sure option was less than that to outcome of a gamble option (*p* < 0.01), and no difference was shown between groups (see Supplement 2).

There was no correlation between gamble choice rate and HR deceleration during the decision-making period in each experimental condition (*r* < -0.33, *n.s.*). Therefore, HR deceleration reflecting trial-by-trial attention to decision-making outcomes did not influence the rate of gamble choice, contrary to the experiential account. Because this result indicated that gamble choice and HR deceleration are independent, we conducted further analyses of these variables separately, as explained in the following section.

### Association between Physiological Parameters and Decision-Making

#### Hypothetical Model

**FIGURE 8:**
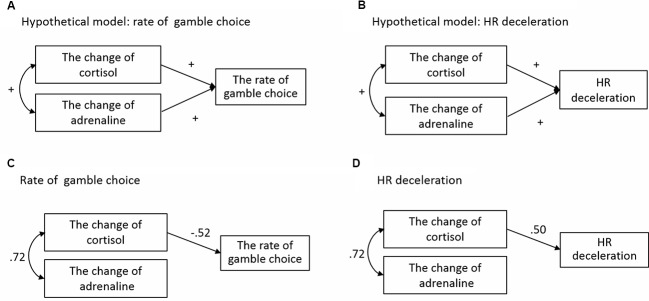
**Structural equation modeling for rates of gamble choice and HR deceleration. (A)** The hypothetical model for rate of gamble choice. **(B)** The hypothetical model for HR deceleration. **(C)** The result of SEM for rate of gamble choice. **(D)** The result of SEM for HR deceleration.

To examine the association between physiological responses caused by acute stress and later decision-making, we conducted structural equation modeling (SEM). We established the hypothetical models shown in **Figures 8A,B**. As described above, because HR deceleration did not affect rates of gamble choice, these were treated as different dependent variables and analyzed separately. In this analysis, we focused on the Equal EV level condition in the Gain domain, in which significant effects of acute stress on decision-making were observed. Magnitudes of HR deceleration to hit and miss outcomes of gamble choices were averaged for each participant and analyzed. Cortisol and adrenaline were used as predictors because previous studies reported that these indexes of the HPA axis and SAM system can affect risk-taking in decision-making and HR deceleration (Putman et al., 2007; Porcelli and Delgado, 2009). For cortisol and adrenaline, change values were calculated by subtracting values at baseline from values immediately after the TSST, and those change values were used as predicted variables.

#### Structural Model

For the rate of gamble choice, we conducted two SEMs. The first SEM (Model 1) was conducted with changes in cortisol and adrenaline as independent variables. Correlation between cortisol and adrenaline was allowed (*r* = 0.47, *p* < 0.05). As a result, a ratio chi-square/df = 18.48, GFI = 0.74 and RMSEA = 0.82 did not indicate adequate overall fit. Therefore, on the basis that effects of glucocorticoid on neural activity are stronger than those of adrenaline (Joëls et al., 2006), we modified the model by deleting a path from adrenaline to the rate of gamble choice. Validity of this model is also suggested by significant correlation between changes in cortisol and the rate of gamble choice (*r* = -0.39, *p* < 0.05) and by non-significant correlation between changes in adrenaline and the rate of gamble choice (*r* = -0.30, *n.s.*) in the Gain domain for participants in the Stress group (*N* = 12). Model 2 (**Figure 8C**) reached balance among statistical requirements and fit the data reasonably well, as indicated by multiple indicators of fit: ratio chi-square/df = 0.39, GFI = 0.98, and RMSEA = 0.00. To evaluate improvement of fit for Models 1 and 2, AIC values (lower indicates better fit) (Schermelleh-Engel et al., 2003) were compared for the two models. The AIC value decreased from 34.48 for Model 1 to 16.74 for Model 2.

Likewise, we conducted two SEMs for HR deceleration. The first SEM (Model 3) was conducted using cortisol and adrenaline as independent variables. Correlation between cortisol and adrenaline was allowed; ratio chi-square/df = 18.48, GFI = 0.74, and RMSEA = 0.82 did not indicate adequate overall fit. For a similar reason to that in the rate of the gamble choice model, we modified the model by deleting a path from adrenaline to HR deceleration (Model 4). Model 4 (**Figure 8D**) fit the data reasonably well, as indicated by multiple indicators of fit: ratio chi-square/df = 0.43, RMSEA = 0.00, and GFI = 0.99. The AIC value comparing improvement of fit strongly decreased from 34.48 for Model 3 to 16.44 for Model 4.

## Discussion

Participants showed fewer risky choices in the Gain domain 2 h after exposure to acute stress, whereas no effect of acute stress on risky choices was observed in the Loss domain. This result replicated a previous finding of domain-specific bias on risky decision-making (reflection effect: greater preference for cautious options in gains rather than losses) observed immediately after exposure to acute stress (Porcelli and Delgado, 2009) and expanded it by showing that such an effect of acute stress can happen even later, when acute physiological stress responses in the HPA axis and the SAM system have disappeared. However, considering the previous finding that risk avoidance was dominant shortly after stress (5 or 18 min) and risk-taking was later preferred (28 min) (Pabst et al., 2013), there might be a complicated fluctuation of preferences for risk in decision-making from risk avoidance to risk-taking and again to risk avoidance along the flow of time after exposure to acute stress. This possibility should be examined more in detail, by multiple manipulating temporal intervals between exposure to acute stress and the decision-making task.

Stress-driven enhancement of preference for a cautious option for a gain was found only in a condition in which the EVs of the sure option and those of the gamble option were identical (Equal EV level); thus, a conflict of choice was the maximum. In conditions in which the EV of either option was greater, participants consistently made rational decisions based on the EV, and no influences of stress were observed. Therefore, it is difficult to attribute the greater preference in stressed participants for cautious gain options to the general impairment of cognitive functions caused by stress (McEwen and Sapolsky, 1995; Vedhara et al., 2000) or to a reduced motivation for monetary reward caused by stress (Ossewaarde et al., 2011). Thus, we suggest that the currently observed results were not just artifacts and that acute stress can have substantially prolonged influences on decision-making. We also infer that the effect of acute stress on risk-taking might be based not on habit action without deliberation, but on strategic, goal-directed action (Schwabe and Wolf, 2011).

Participants exposed to acute stress showed greater magnitudes of phasic HR deceleration just after a feedback signal in the decision-making task than did participants who experienced no stress in the Gain domain, while both groups indicated no differences in their HR deceleration in the Loss domain. This domain-specificity in the prolonged effect of acute stress on HR responses was observed only when an EV of a sure option and that of a gamble option were equal. This result is consistent with the present result of risk avoidance in decision-making. Because HR deceleration following feedback can be interpreted as a sign of attentional orienting (Bradley, 2009; Osumi and Ohira, 2009) and monitoring feedback (Hajcak et al., 2004), this result suggests that risk avoidance in the Gain domain in the Stress group was induced through deliberative processes accompanied by enhancement of attention and monitoring, but not merely through lack of cognitive resources or abandonment of thinking elicited by influences of stress. In addition, HR deceleration was consistently greater when participants chose a gamble option than when they chose a sure option in each condition in each group, suggesting heightened attention to an outcome of gamble (**Figure 1**).

Notably, HR deceleration following feedback did not directly affect decision-making. Thus, acute stress independently affected decision-making and HR responses. In addition, HR deceleration after feedback was sensitive only to domains (Gain vs. Loss), but not to outcomes (Hit vs. Miss). These findings can provide an important suggestion for whether stress effects on decision-making are based on experiential processes or descriptive processes (Buckert et al., 2014). Lack of direct linkage between HR deceleration and risk avoidance and undifferentiated HR deceleration to Hit and Miss outcomes did not support the experience account of stress effects on decision-making, in which reinforcement learning processes according to online evaluations of positive and negative outcomes are hypothesized. Present results are more consistent with the descriptive account, arguing that acute stress can affect decision-making through top–down altering of representations for decision-making structures. In both the Stress and Control groups, the rate of choice for the gamble option was identical when a participant missed a gamble and when he won the gamble (**Table 2**), suggesting that risk avoidance for gains observed in the Stress group did not result from the Lose-shift strategy. Furthermore, values of conditional entropy, an index of dependency of decision-making on previous outcomes, were generally high (*H* > 0.05) and did not differ among experimental conditions (**Table 3**). This result means that Stress group participants did not necessarily depend more on outcomes of immediately previous trials for their decision-making. Therefore, results of these behavioral indices did not support the experiential account, but were more compatible with the descriptive account for risk avoidance observed in the Stress group.

Structural equation modeling results indicated that, specifically in the equal EV condition, both the rate of choice of risky options for gains and HR deceleration were determined by magnitude of cortisol reactivity to acute stress before 2 h. These consistent SEM findings suggest that the cautious shift in decision-making for gains and enhanced attention and monitoring processes reflected by HR deceleration, caused by temporarily separated acute stress, might be produced via involvement of activity in the HPA axis and glucocorticoid receptors in the brain. Secreted cortisol, accompanied by acute stress, can alter functions in glucocorticoid receptors in the brain (Henckens et al., 2010, 2011) and thus can affect psychological processes such as emotional memory (Diamond et al., 2007), selective attention to emotional stimuli (Henckens et al., 2012), and working memory (Henckens et al., 2010). The present study suggests that similar biological processes in glucocorticoid receptors might also affect decision-making under risk. Future research should clarify these detailed biological mechanisms by using neuroimaging and/or pharmacological manipulation.

Limitations of the present study should be noted. First, the sample size of this study is small. However, as described in Section “Results,” most significant effects sizes ($\eta_{p}^{2}$) were within a reasonable range. Additionally, we determined the sample size according to *a priori* analysis to determine statistical power on the basis of Pabst et al.’s (2013) previous finding. Thus, results reported here can be considered mostly acceptable. Nevertheless, considering relatively wide individual differences in present indices, these findings should be replicated with a larger sample before drawing any concrete conclusions. Possible causes of individual difference in indices in this study, for example, personality traits such as trait anxiety (Maner et al., 2007) or genetic factors such as a polymorphism of a serotonin transporter gene (Supplement 3), should be measured and controlled. Additionally, since medication usage of medications was checked only by participants’ self-report, it should be formally tested through urine samples. Second, only male participants were examined to avoid contamination of endocrine variations caused by women’s menstrual cycles. Because previous studies (Preston et al., 2007; van den Bos et al., 2009) reported sex differences in stress effects on risk-taking, the generalizability of present findings must be further examined with samples including both sexes. Third, we did not use the standard TSST control condition (Het et al., 2009) that controls the physical and cognitive load of the task. This policy was adopted to manipulate physiological stress responses in the SAM system and HPA axis as much as possible. Factors of the acute stressor, including the physical and cognitive load-caused effects on decision-making, should be studied further. Finally, other possible physiological pathways mediating effects of acute stress on decision-making should be examined. For example, peripheral pro-inflammatory cytokines, such as interleukin (IL)-1 β and IL-6, increase within 2 h after acute stress (Yamakawa et al., 2009; Brydon et al., 2010). These cytokines reach the brain via leaky regions of the blood–brain barrier and afferent nerve fibers (Raison et al., 2006; Dantzer et al., 2008), and they can affect activity in brain regions such as the amygdala and anterior insula, which are related to affective response (Harrison et al., 2009), leading to modulation of social decision-making (Ohira et al., 2013b).

Despite these limitations, the present study, to our knowledge, has first evidenced that acute stress has prolonged influences on decision-making within hours after exposure to a stressor, leading to a cautious shift in for gains. These findings can provide significant implications for involvement of physiological and somatic factors in decision-making (Ohira, 2010; Ohira et al., 2010, 2013a,b, 2014) not only in an experimental chamber, but also at real financial scenes. Under economic pressures, stock dealers might be too cautious; such effects can affect economic situations.

## Author Contributions

KY and HO designed the study and wrote the paper. These two authors equally contributed to this study. KY conducted the experiment and MM and TI supported the experiment. All authors reviewed the manuscript.

## Conflict of Interest Statement

The authors declare that the research was conducted in the absence of any commercial or financial relationships that could be construed as a potential conflict of interest.
