# Supplementary material for: Prolonged Effects of Acute Stress on Decision-Making under Risk: A Human Psychophysiological Study
Source: Front Hum Neurosci. 2016 Sep 13;10:444. doi: 10.3389/fnhum.2016.00444 (PMC5020085; doi:10.3389/fnhum.2016.00444)
Supplement: Supplementary file 1 [file Table_1.DOCX]

Supplement 1. Attribute of gambling option.

(a) Gain domain.

| 40% (Keep = + 200 yen)  Amount of money = 0 yen | |  | 60% (Keep = + 360 yen)  Amount of money = 0 yen | |
| --- | --- | --- | --- | --- |
| win | lose | EV level | win | lose |
| + 700 | 0 | large | + 800 | 0 |
| + 600 | 0 |  | + 700 | 0 |
| + 500 | 0 | equal | + 600 | 0 |
| + 300 | 0 | small | + 500 | 0 |
| + 200 | 0 |  | + 400 | 0 |

(b) Loss domain.

| 40% (Keep = − 300 yen)  Amount of money = 500 yen | |  | 60% (Keep = −240 yen)  Amount of money = 600 yen | |
| --- | --- | --- | --- | --- |
| win | lose | EV level | win | lose |
| + 200 | −500 | large | + 200 | −600 |
| + 100 | −500 |  | + 100 | −600 |
| 0 | −500 | equal | 0 | −600 |
| −100 | −500 | small | - 100 | −600 |
| −200 | −500 |  | - 200 | −600 |
