# Supplementary material for: Prolonged Effects of Acute Stress on Decision-Making under Risk: A Human Psychophysiological Study
Source: Front Hum Neurosci. 2016 Sep 13;10:444. doi: 10.3389/fnhum.2016.00444 (PMC5020085; doi:10.3389/fnhum.2016.00444)

Supplement 2. The HR deceleration to the outcome in decision-making.

There was a significant interaction between Group, Domain, and Feedback (Hit vs Miss vs Keep) (*F* (1.33, 30.67) = 10.92, *p* < 0.01, *η^2^* = 0.32). Post hoc comparisons (*p* < 0.01) revealed that the HR deceleration to the outcome of keep option was less than that to the outcome of gamble option (*p* < 0.01), and no difference was shown in between the groups (*n.s.*).


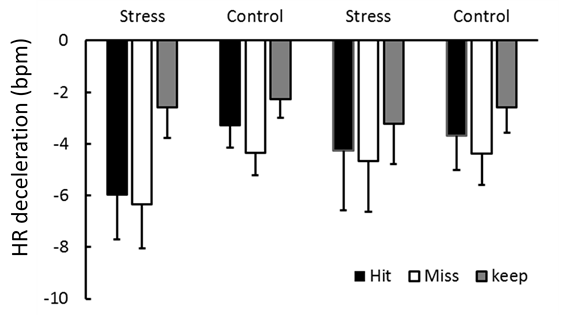

Supplement: Supplementary file 2 [file Data_Sheet_1.DOCX]
