# Supplementary material for: Prolonged Effects of Acute Stress on Decision-Making under Risk: A Human Psychophysiological Study
Source: Front Hum Neurosci. 2016 Sep 13;10:444. doi: 10.3389/fnhum.2016.00444 (PMC5020085; doi:10.3389/fnhum.2016.00444)

Supplement 3. Multiple-group structural equation modeling for rates of gamble choice each 5HTT genotypes.

We examined whether the inter-individual difference of association between stress reactivity and decision-making in the Stress group of the present study was explained by a genetic factor, the serotonin (5-hydroxytriptamine, 5HT) transporter (5HTT) gene polymorphism, using the multiple-group structural equation modeling (SEM). The method of genotyping was described elsewhere (Yamakawa et al. 2015). It was previously demonstrated that individuals with homozygous short (S) alleles of the 5HTT gene-linked polymorphic region (5HTTLPR) (the SS genotype) showed greater activation of stress-related brain regions (Ohira et al., 2009) and secretions of cortisol (Gotlib et al., 2008, Yamakawa et al., 2015) in response to acute stressors than did individuals with S and Long (L) alleles (the SL genotype) and homozygous LL alleles (the LL genotype). We observed that the rate of choice of risky options for gains was negatively determined by the magnitude of cortisol reactivity to acute stress before 2 hours in participants with the SS genotype (N = 6), but cortisol reactivity to acute stress did not affect choices of risky options in participants with heterozygous S and L alleles (N = 6). This result is reasonable considering that individuals with homozygous S alleles consistently showed greater stress reactivity in cortisol secretion (Gotlib et al., 2008, Yamakawa et al., 2015). Though this finding is preliminary due to the small sample size, the result of the multiple-group SEM suggests that possible sources of individual differences of stress effects on decision-making are gene polymorphisms, including 5HTTLPR (ratio chi-square/df = 0.71, GFI = 0.96, and RMSEA = 0.00).


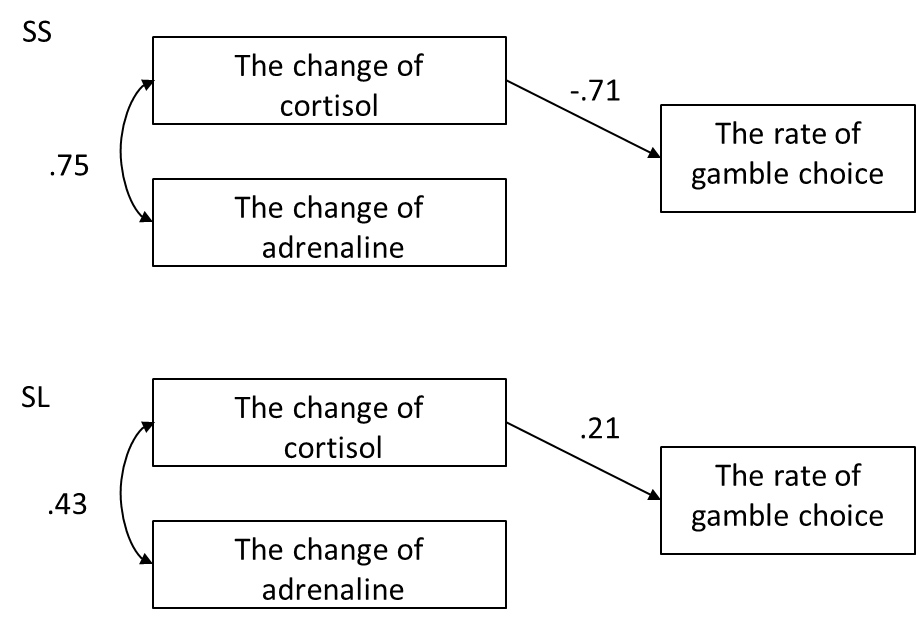

Supplement: Supplementary file 3 [file Data_Sheet_2.DOCX]
